# Lipid-Based Gels for Delivery of 3-O-Ethyl L-Ascorbic acid in Topical Applications

**DOI:** 10.3390/pharmaceutics16091187

**Published:** 2024-09-07

**Authors:** Noèlia Loza-Rodríguez, Aina Millán-Sánchez, Mireia Mallandrich, Ana Cristina Calpena, Olga López

**Affiliations:** 1 Department of Chemical and Surfactant Technology, Institute of Advanced Chemistry of Catalonia (IQAC-CSIC), C/Jordi Girona 18-26, 08034 Barcelona, Spain; aina.millan@iqac.csic.es; 2 Bicosome S.L., C/Jordi Girona 18-26, 08034 Barcelona, Spain; 3 Department of Pharmacy and Pharmaceutical Technology, Faculty of Pharmacy, University of Barcelona, C/Joan XXII 27-31, 08028 Barcelona, Spain; mireia.mallandrich@ub.edu (M.M.); anacalpena@ub.edu (A.C.C.)

**Keywords:** 3-O-ethyl ascorbic acid, release, skin permeation, drug delivery, hydrogel, bigel, lipids

## Abstract

This study explores the incorporation of 10% 3-O-ethyl L-ascorbic acid (ETVC), a derivative of vitamin C, into two lipid gel systems: a hydrogel (HG) consisting exclusively of lipids and water and a bigel (BG) combining the hydrogel with an oleogel made from olive oil and beeswax. We investigated the ETVC release profiles from both materials using synthetic membranes and measured their permeation through porcine skin in vitro. Additionally, the interaction of these lipid gel systems with the stratum corneum (SC) was determined. Results from the release study indicate that the BG exhibited slower ETVC release compared to the HG. The permeation experiments showed that the presence of lipids in the formulations enhanced ETVC retention in the skin. The HG delivered a higher amount to the SC, while the BG achieved greater retention in the epidermis. This difference is attributed to the different lipophilic nature of each material. The structural analysis of SC lipids revealed that the organization of surface lipids remained unaltered by the application of the gels. Finally, an in vitro efficacy test in porcine skin using methylene blue indicated that our ETVC gels exhibited antioxidant activity. These findings provide valuable insights into the potential of lipid-based gels for topical applications.

## 1. Introduction

Vitamin C is a potent antioxidant molecule used in dermatology to treat and prevent changes associated with photoaging, hyperpigmentation and collagen synthesis [1,2,3,4,5,6,7]. It has been observed that aged and/or photoaged skin has a lower vitamin C concentration than young skin due to prolonged exposure to oxidative stress from pollutants and UV irradiation [3,8,9,10,11,12,13]. Therefore, the topical application of vitamin C is one of the most used strategies to supply this vitamin in the treatment of numerous dermatological problems [14,15]. L-Ascorbic acid (AA) is the chemical name of vitamin C and is useful in medical practice, as humans cannot synthesize it [5]. However, AA poses challenges for skin delivery and formula stability in cosmetic and pharmaceutical products due to its rapid degradation.

**Figure 1:**
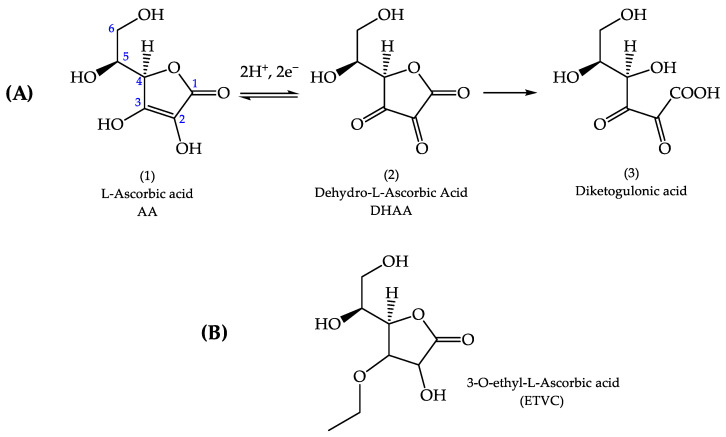
Oxidation process of L-ascorbic acid (**A**). Chemical structures of L-ascorbic acid (**1**), dehydro-L-ascorbic acid (**2**), diketogulonic acid (**3**) and 3-O-ethyl-L-ascorbic acid (**B**) [16].

AA degradation is triggered by its ionization in aqueous solution. On exposure to temperature, light, dissolved oxygen, catalytic amounts of metal ions, etc., AA is oxidized to dehydro ascorbic acid (DHAA) (see Figure 1A(2)), which is too electrophilic to survive more than a few milliseconds in aqueous solution and decays irreversibly to diketogulonic acid (see Figure 1A(3)), leaving a brown color [16]. For optimal penetration to the epidermis, aqueous formulations of AA (including its sodium salt) must be at a pH < 3.5, below the pKa limit (pKa 4.2). Therefore, the ionic charge is removed, and the molecule is transported well across the SC [16,17,18]. However, skin pH is around 5.5, and topical formulas with a pH below 3.5 may irritate the skin. To solve this problem, various research groups have proposed diverse strategies to slow down the oxidation of AA and explore its antioxidant activity. These approaches include encapsulating the molecule in emulsions and nanoemulsions, liposomes, inorganic nanocapsules and acid serums. Although most of these encapsulation strategies have many advantages, including biocompatibility, hydrophilic drug delivery and low toxicity, depending on the components used and the material microstructure, they may show limitations as drug carriers, such as poor stability, the possibility of rapid drug release, limited hydrophobic delivery, low mechanical strength or not being biomimetic enough. Additionally, to avoid vitamin C degradation, AA derivatives have been synthesized, and different formulations with redox partners and stabilizers such as vitamin E have been tested [14,16,17,19,20,21,22,23,24,25,26,27,28].

3-O-ethyl-L-ascorbic acid (ETVC) is a derivative of AA widely used in topical products [17,27,28,29]. The ethyl group modification (see Figure 1B) protects the 3-OH group from ionization, thereby avoiding the rapid oxidation of the molecule. Iliopoulos et al. [17] reported the pKa of ETVC to be 7.72 ± 0.01. By formulating products with ETVC at pH 5.5 (matching skin pH), the molecule remains uncharged, facilitating its transport across the SC and stabilizing the molecule without lowering the pH to the extent required for AA (below 3.5). These studies have contributed to advancements in the field, yet ongoing efforts are essential to develop drug delivery systems capable of maintaining AA stability and ensuring the permeation to the skin.

Our research group has recently developed two gel-like materials, a lipidic hydrogel (HG) [30,31,32,33] and a new biocompatible lipid-based bigel (BG) [34]. Loza-Rodríguez et al. [34] reported these two materials to show interactions with the skin, which enhanced the penetration of hydrophilic and lipophilic fluorescence probes into the cutaneous tissue. In addition, the capacity of these HG and BG materials to protect pure AA from degradation upon temperature and sunlight [35] compared to other materials [28] was reported.

In this work, we incorporated ETVC into the HG and the BG to investigate release kinetics and permeation in dialysis membranes and porcine skin. Additionally, the interaction of these lipid gel systems with the SC was assessed to evaluate any possible modifications in the structure and organization of the SC lipids after gel application. Finally, an in vitro efficacy test was conducted to evaluate the antioxidant capacity of our gels with ETVC when applied to porcine skin. Our gels are composed only of lipids and water. The literature described hydrogels and bigels with different compositions [36,37,38,39,40,41,42,43,44,45,46,47,48], as well as different strategies for encapsulating ascorbic acid such as emulsions, liposomes, acid serums and hydrogels [22,24,28,29,49,50,51,52]. However, these incorporate polymers, resins, skin permeation enhancers or chelating agents. There are also studies on the release of ETVC or vitamin C using different solvents, enhancers, serums and polymeric hydrogels [17,18,22,27,29,37,52,53,54,55], and there are numerous market products with vitamin C, but none involve lipidic gels without polymers, skin permeation enhancers or resins. The HG used in this study is a colloidal lipid gel formed by the aggregation of phospholipid vesicles under dilute conditions [30,31,32,33]. On the other hand, the BG is a combination of the aforementioned HG and an oleogel (OG) composed of olive oil (OO) and beeswax (BW) [34,35]. Both gels are only prototypes, but all the components confer high biocompatibility, mimicking skin’s own components, which could enhance interaction and skin permeation. Moreover, the BG is a two-phase system with solid-like characteristics, enhancing the stability of the formula compared to that of emulsions and liposome solutions [35,36,51,56]. Their microscopic structure, rheological behavior and soft nature [35] make these materials potential candidates for topical applications.

## 2. Materials and Methods

### 2.1. Materials

3-O-Ethyl ascorbic acid (ETVC) was distributed by Safic-Alcan Especialidades, S.A.U. (Spain), and supplied by Corum Inc. (Taipei, Taiwan). Hydrogenated soy phosphatidylcholine (HSPC; Phospholipon^®^ 90H) and 1,2-dioleoyl-3-trimethylammonium-propane (DOTAP) were supplied by Lipoid GmbH (Ludwigshafen, Germany). Extra virgin olive oil was obtained from Aceites del Sur—Coosur, S.A. (Vilches, Jaén, Spain), and beeswax was purchased from EsentialArôms Dietéticos Intersa, S.A. (Alcarràs, Lleida, Spain). Chloroform was supplied by Carlo Erba Reagents S.A.S. (Val de Reuil, Paris, France) and α-tocopherol acetate by Guinama S.L.U (La pobla de Vallbona, Valencia, Spain), and purified water was obtained from an ultrapure water system, Milli-Q plus 185 (Millipore, Bedford, MA, USA). Methylene blue was purchased from Thermo Fisher Scientific (Cornellà de Llobregat, Spain). Methanol for liquid chromatography and sodium dodecyl sulfate were supplied by Sigma Aldrich (Merck KGaA, Darmstadt, Germany).

### 2.2. Gel Preparation

#### 2.2.1. Hydrogel

The HG was prepared following the film hydration method, according to the literature [31,33,34,35]. In summary, HSPC and DOTAP were solubilized with chloroform in a round-bottom flask and slowly evaporated with a rota-evaporation system to obtain a lipid film. Afterwards, the film was hydrated with an aqueous solution of ETVC using an ultrasound bath at 25 °C. Then, a temperature cycle was performed in a closed vial: first, it was frozen at −20 °C for 3–4 h; then it was heated at 70 °C for 10 min; and finally, the solution was left to cool at 5 °C in the fridge to obtain gelation. Gelation was confirmed by the absence of gravitational flow when the test tubes containing the HGs were inverted, the so-called “inversion test”. Two HGs were prepared, one with 25% ETVC and the other with 10% ETVC. The most concentrated HG was used to form the BG. The final composition of each gel is detailed in Table 1.

#### 2.2.2. Bigel

Initially, the OG and HG were prepared as separate materials. The OG formulation involved weighing and mixing of OO, BW and tocopherol, followed by heating at 70 °C to achieve a fluid material. To obtain the final BG 10% ETVC, the OG was mixed with the HG 25% ETVC at 70 °C using 40% HG and 60% OG. The mixture was stirred for 10 min at 1100 rpm with a magnetic bar and then allowed to cool to 25 °C. Gelation was confirmed by the inversion test, and the absence of phase separation indicated the correct formation of the BG.

All the experiments performed in this study were conducted with freshly prepared gels. In addition, after 7 months at 8 °C, all the gels had the same visual appearance, texture and organoleptic properties as freshly prepared gels, without any sign of contamination.

### 2.3. In Vitro Release Study

The release study of ETVC incorporated into gel formulas was executed in Franz cells (Lara-Spiral, Courtenon, France), which were kept at 37 ± 0.5 °C to mimic skin in vivo conditions by means of a circulating water bath (Julabo Labortechnik GmbH, Seelbach, Germany). A magnetic bar was introduced into the receptor chamber to maintain the receptor fluid under constant stirring. MilliQ water was used as the receptor fluid. For this study, methylcellulose membranes (Dialysis tubing Membra-Cel™ cellulose, Flatwidth 34 mm, MWCO 14 000 Da 30 m, Carl Roth, Germany) were hydrated for 24 h, placed between donor/receptor compartments and sealed with parafilm. Then, 50 µg of each sample (water solution 10% ETVC, HG 10% ETVC, BG 10% ETVC) was added to the donor phase membrane (application area of 1.86 cm^2^) to maintain sink conditions. Aliquots of RF (320 µL) were collected at selected times, being replaced with the same amount of RF. ETVC quantification was performed using an HPLC HP1260 Infinity II instrument (Agilent Technologies, Santa Clara, CA, USA). The apparatus consisted of a binary pump (G7112B), autosampler (G7129A) and DAD detector (G7117C). The system was operated using the software OpenLab CDS 2.1. The mobile phases were 10% MeOH and 90% MilliQ water with a flow of 1.2 mL/min. The column was a Zorbax Eclipse Plus C18. The analysis was performed at 25 °C. A total of 10 µL of the sample was injected, and the signal was detected at ʎ = 244 nm. To quantify the final ETVC concentration, a calibration curve was constructed using a standard solution of ETVC. Two stock solutions were prepared, and subsequent dilutions were mixed accordingly to achieve the following concentrations: 0.01, 0.1, 1, 5, 50, 200, 500 and 1000 ppm. Two replicates of each vial were injected. 

The analytical methodology was previously validated in terms of the calibration curve, limit of detection, limit of quantification and precision. According to Pullar et al. [3], to prepare ascorbic acid derivatives for the purpose of topical applications, one needs to ensure the stabilization of the molecule from oxidation and also overcome the significant challenge of skin penetration. In addition, derivatives must be converted to ascorbic acid in vivo in order to be effective [3]. The analytical method described in this study was only for ETVC content determination, in which the only peak observed was that of ETVC. In all chromatograms, no other peaks corresponding to ascorbic acid AA were detected, even though the final antioxidant effect was due to ETVC conversion into AA in the skin.

Further, to assess the ETVC release through methylcellulose membranes, the release data obtained were examined using a hyperbole kinetic model, which was the best based on the highest correlation coefficient. The model was obtained using the software Graphpad Prism 9. Once the model was defined, it was possible to calculate the velocity constant (*Kd*) and the % maximum concentration (*Bmax*) using Equation (1):

(1) $$Y=\frac{Bmax\cdot X}{Kd+X}$$

### 2.4. In Vitro Skin Permeation and Kinetics Study

Pig skin from the back of Large White pigs weighing approximately 15 Kg was obtained from the Faculty of Veterinary of Autonomous University of Barcelona. The skin was removed from the back of the pig few hours after the animal was sacrificed for medical experimentation. The protocols used were approved by the Ethical Commission of Animal and Human Experimentation (Spanish Government) under the auspices of the Ethical Commission of the Autonomous University of Barcelona. The bristles were removed carefully with an animal clipper, and the skin was washed with distilled water. The excised skin was dermatomed to 500 ± 50 µm thickness (Dermatome (GA620, Aesculap, Tuttlingen, Germany). Discs of dermatomed skin were obtained with an iron punch (2.5 cm inner diameter). They were vacuum packed and stored at −20 °C until use. One hour prior to the experiments, the skin was thawed at room temperature.

The Franz cells utilized in this experiment were identical as the ones in the previous release studies, but in this case, porcine skin was used. The application area was 1.86 cm^2^. A magnetic stirring bar was introduced into the receptor chamber. The skin disc was mounted with the SC side up in the Franz cell. The receptor chamber was filled with MilliQ water as the receptor fluid. Previous experiments were conducted using PBS and water as the RF to optimize the final method for assessing the stability of the molecule throughout the analytical process. These experiments demonstrated that ETVC exhibited higher stability when MilliQ water was used. Franz cells were kept at 37 ± 0.5 °C by means of a circulating water bath (Julabo Labortechnik GmbH, Germany) to ensure that the surface skin was maintained at 32 ± 1 °C. The integrity of each skin sample was checked by determining the transepidermal water loss (TEWL) using a Tewameter TM210 (Courage-Khazaka, Köln, Germany) one hour after the cells were prepared.

The experiment was initiated by applying gently 100 µg of the samples (water solution 10% ETVC, HG 10% ETVC, BG 10% ETVC) with a round-ended microspatula for gels and a micropipette for water solution. A control cell was also used with the application of only 100 µL of water.

For the kinetics study, aliquots of RF (320 µL) were collected at selected times and replaced with the same amount of MilliQ water. After 24 h of exposure time, the test formulation remaining on the skin surface was removed with a specific wash, as described in Rubio et al. [57]. First, the skin was washed with 0.5 mL of sodium dodecyl sulfate (SDS) solution (at 0.5% *w*/*w*) and then twice with 0.5 mL of MilliQ water. After that, the skin surface was dried with a cotton swabs. All washing aliquots (SDS and water), tips of the micropipette, all cotton swabs and the top of the cell were pooled, constituting the fraction of the active compound remaining on the surface. To complete the final wash solution, 10 mL of MeOH/H_2_O 1:1 solution was added. Afterwards, the RF was removed from the receptor compartment and brought up to 5 mL with MilliQ water in a volumetric flask. The SC of the treated skin area was removed by 8 successive tape strippings using adhesive tape (D-Squame^®^, CuDerm Inc., Dallas, TX, USA). Then, the viable epidermis was separated from the dermis after heating the skin at 80 °C for a few seconds. To extract ETVC from the SC adhesive tapes, 4 mL of MeOH was added, and to extract from the epidermis and dermis, 1 mL was added respectively. All solutions, except for the RF that was injected in the HPLC equipment on the same day, were kept overnight and protected from light. The next day, samples were shaken for 2 h at 32 °C with an Unitronic Vaivén C. JP SELECTA (Scharlab, Sentmenat, Barcelona, Spain) and sonicated for 10 min. Before analytical determination by HPLC, all samples were filtered using nylon syringe filters with a 0.22 µm pore size before being injected. For wash BG samples (from the remaining BG on the skin surface), two nylon filters were needed. The analytic method was the same as that described in Section 2.3.

The permeation rates of ETVC at a steady state (J, μg/cm^2^·h) were estimated from the slope of the linear part of the plot of cumulative permeated ETVC across the skin area vs. time, and lag time (*Tl*) was estimated by the x-intercept. This parameter (*Tl*) was defined by the time it took for the ETVC to pass through the skin and reach the receptor fluid. *Kp*, *P*1 and *P*2 parameters were calculated according to Equations (2)–(4) [58,59,60], where Escribano et al. [60] reported that *C*_0_ is the concentration of ETVC in the initial formulations, *Kp* is the permeability constant and, according to Okamoto et al. [61], *P*1 and *P*2 are parameters directly related to the membrane/donor phase partition coefficient and diffusion coefficient, respectively.

(2) $$Kp=J/C_{0}$$

(3) P2=(1/6)Tl

(4) P1=P2/Kp

### 2.5. Skin Surface Lipid Structure

Grazing incidence small-angle X-ray scattering (GISAXS) measurements were carried out using a SAXS/WAXS S3-MICRO (Hecus X-ray Systems GmbH, Graz, Austria) coupled to a GENIX-Fox 3D X-ray source (Xenocs, Grenoble, France) with a wavelength corresponding to the CuKα line (1.542 Å). The linear detector used was a PSD 50 M instrument (Hecus, Graz, Austria), temperature control was performed via a Peltier TCCS-3 instrument (Hecus, Graz, Austria) (precision better than ±0.1 °C) and the sample-to-detector distance was 268 mm.

For sample preparation, 10 mg of water, HG and BG were applied on the skin with a spatula with an area of application of 0.5 cm^2^. After 16 h of incubation at 37 °C in a wet environment, the excess of material on the surface was removed gently with filter paper, and the skin was washed with 10 µL of water. The sample was cut and mounted by deposition on oxidized silicon 111 cut-plane wafers. The wafers were oriented parallel to the X-ray beam by a stepping motor with a resolution of 0.1°. A homemade accessory was used to maintain the humidity in skin samples by providing a humid atmosphere during the experiment, and this also permitted the alignment of the sample between 0.5° and 0.25° of the incident angle. Humid air at 32 °C was blown into the sample cell at 90–99% relative humidity.

The scattering intensity I (in arbitrary units) was measured as a function of the scattering vector *q* resulting from a photon of wavelength *λ* scattering off the sample at an angle 2*θ*, as described by Equation (5) [62]:

(5) $$q=\frac{4\pi sin\theta }{\lambda }$$

Subsequently, the Bragg’s distance was calculated using Equation (6) [62]:

(6) $$D_{Bragg=}\frac{n2\pi }{q}$$

In a lamellar structure, the various peaks are situated at equidistant positions, where *Qn* in Equation (7) represents the position of the nth-order reflection [62].

(7) $$Qn=\frac{2\pi n}{d}$$

The data analysis and acquisition of *q* values were conducted using ORIGIN Pro 2019 software.

### 2.6. In Vitro Skin Antioxidant Efficacy

The qualitative antioxidant capacity of water solution 10% ETVC, HG 10% ETVC and BG 10% ETVC to reduce methylene blue to colorless leucomethylene blue [63] was analyzed using in vitro pig skin. Skin from the back of Large White pigs was obtained from the Faculty of Veterinary of Autonomous University of Barcelona, as explained in Section 2.4. The excised skin was dermatomed to 500 ± 50 µm thickness (Dermatome (GA620, Aesculap, Tuttlingen, Germany) and was vacuum packed and stored at −20 °C until use. One hour prior to the experiments, the skin was thawed at room temperature. Afterwards, skin samples were cut into 2 cm^2^ sections and placed with the SC facing up into 6 different petri dishes in a wet environment to keep the bottom of the tissue moist. Then, 50 µL of methylene blue 0.01% was added onto the skin surface and incubated at 32 °C for 4 h. Skin samples were washed and dried using filter paper on the SC. After this procedure, 30 mg of either gel samples or water solution of ETVC was applied onto the skin with an area of 1 cm^2^, and water was applied to another skin sample as the control. The samples were incubated again during 1 h. Images were recorded right after administering the formulations and after the treatment using a Samsung Camera with a resolution of 1884 × 4080 and F/1,8 aperture.

### 2.7. Statistical Analysis

For in vitro release studies (Section 3.1), at least three replicates for each sample were analyzed. Values are expressed as the % average of weight ETVC released/weight ETVC applied ± standard error. The differences between materials in the release of ETVC at each time point were analyzed using a two-way ANOVA (or mixed model) with the Greenhouse–Geisser correction. In addition, kinetic parameters differences between Bmax and Kd values were determined with Brown–Forsythe and Welch one-way ANOVA tests.

Regarding the in vitro skin permeation studies of ETVC, at least three replicates for the skin kinetic studies (Section 3.2.1) and at least six replicates for the permeation studies (Section 3.2.2 and Section 3.2.3) were analyzed. In Section 3.2.1, all figures are represented as the % average of ETVC diffused through the porcine skin corresponding to the % average weight of ETVC released in the RF/weight ETVC applied to the skin ± standard error. The differences between materials in the skin kinetic release (Section 3.2.1) of ETVC in each time point were analyzed using a two-way ANOVA (or mixed model) with the Greenhouse–Geisser correction. In addition, the linear regression to calculate the skin kinetic parameters was performed, and differences between *J*, *Tl*, *Kp*, *P1* and *P2* were analyzed with a one-way ANOVA non-parametric test with Graphpad Prism 9 (Boston, MA, USA).

Total skin permeation and receptor fluid values (Section 3.2.2 and Section 3.2.3) are represented as the % average of ETVC diffused through the porcine skin based on the total amount of ETVC that permeated into the skin and RF, corresponding to the % average of ETVC released in the different skin layers/ETVC found in the skin + RF ± standard error. Significant differences were analyzed using an unpaired t-test between the skin and FR for each material and Brown–Forsythe and Welch one-way ANOVA tests for ETVC differences between materials. The ETVC permeation in skin layers for each material (Section 3.2.3) was analyzed with Brown–Forsythe and Welch one-way ANOVA tests. Furthermore, differences in the penetration of ETVC in the same skin layer between materials were analyzed using a two-way ANOVA (or mixed model) with the Greenhouse–Geisser correction.

All data were analyzed using GraphPad Prism 9. Statistical significance was determined by *p* values < 0.05. 

## 3. Results and Discussion

### 3.1. In Vitro Release Studies

**Figure 2:**
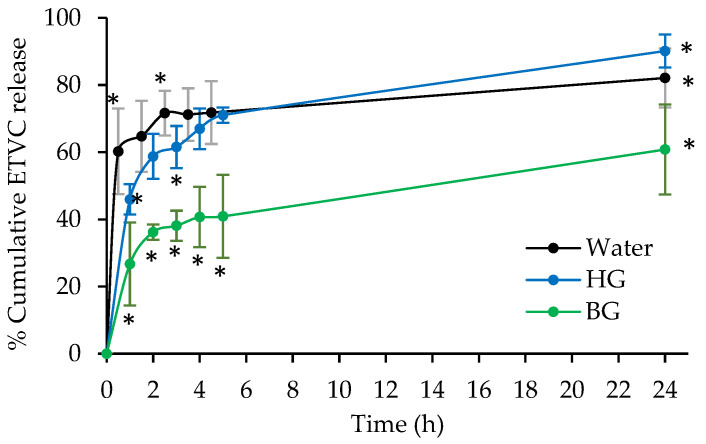
ETVC in vitro release profile for the water solution, HG and BG, expressed as % mean of weight ETVC released/weight ETVC applied ± SD (n = 3). Significant differences between materials at different time points are represented as asterisks (*).

**Figure A1:**
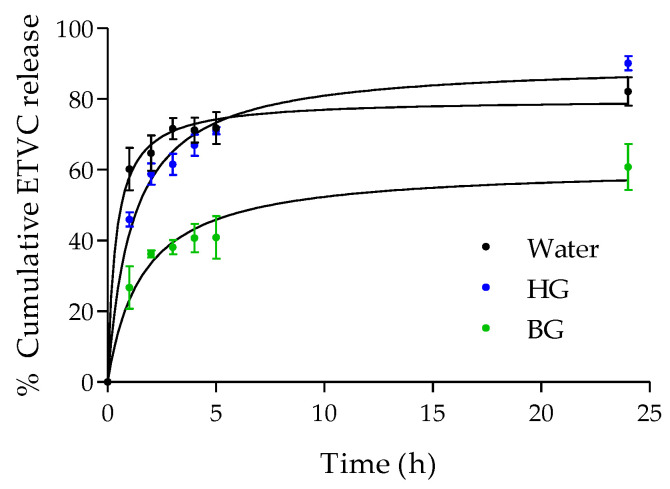
In vitro kinetic results represented with the hyperbole model correlation (see Equation (1)), adjusted with GraphPad Prism 9. The graph represents the values as mean ± standard error (not SD).

The in vitro release studies conducted with diffusion membranes provide us with information about the behavior of ETVC release for each lipid system. All the experiments performed in this study were conducted with freshly prepared gels. Profiles representing the cumulative percentage of ETVC released within 24 h are shown in Figure 2. Samples were collected every hour during the first 5 h, with the final sample taken at 24 h. The percentage data were calculated based on the total amount of product applied to the skin and expressed as weight ETVC released/weight ETVC applied. All data values represented in Figure 2 and the statistical analyses are presented in Appendix A, Table A1 and Table A2, respectively. The release values in Figure 2 were correlated with a kinetic model. In this case, ETVC profiles followed a hyperbolic diffusion model for the three systems studied (see Appendix A, Figure A1). The velocity constant (*Kd*) and the maximum concentration release values (*Bmax*) are represented in Table 2. Lower *Kd* values mean higher velocities (see Equation (1)). These kinetic parameters provide insights into the rate and final concentration of drug release, influenced by the encapsulation method and formulation ingredients.

The ETVC release depended on the nature of the delivery system. In Figure 2, the % ETVC released by the aqueous solution and HG is similar, although higher values are observed for water, especially during the first and third hours of the experiment, reaching a 60% and 71%, while the HG only showed 45% and 61% release at the same time points, respectively. However, after 5 h, a similar percentage of drug release is detected for both HG and water, with 71% of ETVC released. After 24 h, even though the velocity constant (*Kd*) is higher for water solution (see Table 2), the maximum ETVC released (*Bmax*) is achieved by the HG, with 90% released, followed by the water solution with 82%.

The BG exhibited the lowest percentage of ETVC release throughout the experiment, with significant differences observed at all time points (*p* < 0.05) when compared to the values for water and HG. After 24 h, BG released 60% ETVC. According to kinetic parameters, BG had the lowest velocity constant and the lowest % ETVC released (*Bmax*) compared to water and HG (see Table 2).

These differences between the water, HG and BG formulations can be attributed to the structural characteristics and encapsulation mechanisms of each material. HG consists of lipid vesicles that form branched aggregates in an aqueous medium [30,31,34]. Given the solubility of ETVC, this molecule is distributed in the aqueous part, both inside and outside of the lipid vesicles that form the three-dimensional structure [34]. This encapsulation explains the higher *Kd* value compared to water, indicating a slower release rate of ETVC from the HG. HG has a fast initial release related to the free ETVC in the water, followed by a sustained release of ETVC encapsulated inside the vesicles, resulting in a higher % ETVC release from HG compared to water after 24 h. Alhelal et al. [64] observed a similar behavior with a hydrogel loaded with solid–liquid nanoparticles, where the initial fast drug release was attributed to the drug present outside the encapsulation, followed by a delayed release over 24 h. 

Conversely, the BG, in which the HG is incorporated into a continuous OG matrix, as previously reported by Loza-Rodríguez et al. [34], seems to exhibit a different release behavior. In this system, ETVC is encapsulated within the HG phase (distributed in the water both inside and outside of the vesicles), and the HG, in turn, is trapped in the OG matrix, so the release mechanism can involve two events: (1) the ETVC travels from HG to OG, and then it is released; and (2) the ETVC is released directly from HG. This would occur because the mechanical application of the BG on a surface promotes the separation of the BG components and facilitates the direct contact of the HG with the application surface. Considering the high water solubility of ETVC, the second mechanism suggested would be the most probable. Therefore, the BG exhibits a slower release profile compared to the HG. After 24 h, the BG had only released 60% ETVC, whereas the HG had already released 90% by that time. Our results align with those from Khan et al. [65] and Maji et al. [66], where the double encapsulation of microparticles and transferosomes into a BG, respectively, resulted in a slower release compared to the single drug encapsulation in the BG itself. 

From a therapeutic perspective, a rapid release pattern is likely advantageous for initiating treatment effects. Subsequently, the delayed release of the remaining medication helps sustain the therapeutic dose, reducing the need for multiple administrations [64]. 

### 3.2. In Vitro Skin Permeation Studies

#### 3.2.1. Skin Kinetics

**Figure 3:**
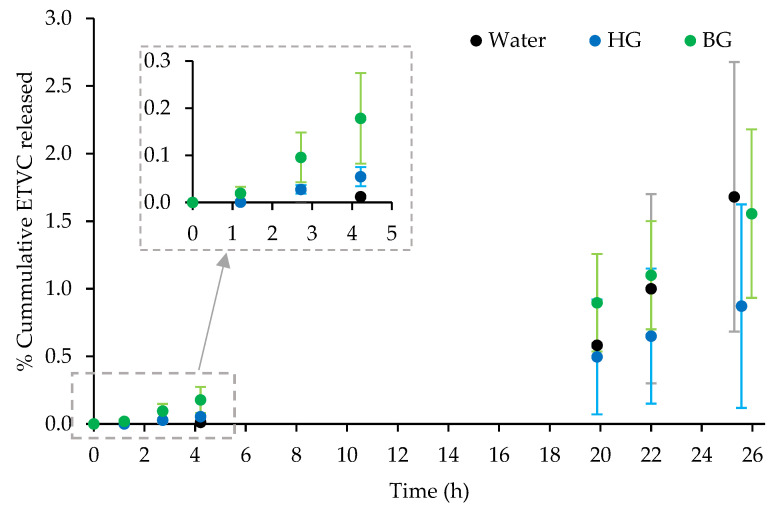
In vitro ETVC kinetic profile through porcine skin for the water solution, HG and BG, expressed as % mean (weight ETVC released in the RF/weight ETVC applied to the skin) ± SD (n = 3). An inset graphic from 0 to 4 h is represented to highlight the significant differences in the performance of the BG.

The in vitro kinetic studies conducted with porcine skin provide us with information about how the different materials can influence the transdermal flux of the drug as well as the residence time in the biological tissue. This allows us to compare the biopharmaceutic behavior and the physicochemical characteristics of the HG and the BG. Profiles representing the percentage of total ETVC released through the skin within 24 h are shown in Figure 3. The percentage data were calculated based on the total amount of product applied to the skin expressed as weight ETVC released/weight ETVC applied. All data values represented in Figure 3 and the statistical analyses are presented in Appendix B, Table A4, Table A5 and Table A6, respectively.

The permeation parameters of ETVC are represented in Table 3. The permeation rate at the steady state (*J*, μg/cm^2^·h) was estimated from the slope of the linear part of the plot of cumulative permeated ETVC across the skin area (µg/cm^2^) vs. time (h). J is related to the permeation flux of ETVC once it arrives to the fluid receptor and reaches a steady rate. The lag time (*Tl*) is the X-intercept point with the linear regression. This is related to the time that takes for the ETVC to pass through the skin. *Kp, P1* and *P2* parameters were calculated according to Equations (2)–(4) respectively. *Kp* is the permeability constant, directly proportional to *J* (see Equation (2)), and a higher value means a higher permeability. *P1* is related to the partition coefficient of the ETVC vehicle skin, and a higher value means that ETVC will have a higher tendency to stay in the vehicle than to pass through the skin. *P2* is related to the diffusion coefficient, and both are directly proportional to *Kp* (see Equation (4)) [58,59,60,61]. All statistical analyses are presented in Appendix B Table A5 and Table A6.

To understand these results, it is essential to consider how both the ETVC and the systems (HG and BG) interact with the skin. Independent of the systems, ETVC will enter the skin and will be retained for a period (lag time, *Tl*) before reaching the receptor fluid. Once it reaches the receptor fluid, the permeation kinetics follow a steady state. Table 3 shows that when ETVC is applied in aqueous solution, the values of *J, Tl, Kp, P1* and *P2* are higher than when it is applied with HG and BG. However, significant differences were only encountered between *Kp* and *P1* for HG and the water solution. There were no differences between HG and BG kinetic parameters. For the ETVC aqueous solution, the higher *Kp* value means that once the release to the receptor fluid begins, the rate of permeation is faster when ETVC is applied in water than when it is applied with the HG. ETVC, as a hydrophilic molecule, will face certain difficulty in passing through the SC. Considering that hair follicles, sweat ducts and sebaceous glands are the usual skin permeation pathways for hydrophilic free molecules [67], the ETVC could follow these pathways when applied in aqueous solution. Our hypothesis is that once the ETVC reaches the epidermis, as this has a much higher water content compared to the SC, the permeation will be faster and the tendency will be to permeate through the skin, not being retained in the skin layers (higher *Kp*). Furthermore, *P1* values between HG and the water solution indicate a higher tendency for ETVC to pass through the skin in the presence of HG compared to the water solution. Since ETVC is a hydrophilic molecule, it is more likely to remain in the water rather than pass through the SC. By including ETVC in the HG, the interaction with the skin is improved due to a higher lipid content [68].

The diffusion tests through the skin provide information about how the system and the drug interact with the skin. On the other hand, the in vitro release study of ETVC through membranes (Section 3.1) reports how the system itself releases the drug. This second test will depend on the nature of the drug and the nature of the system. Water and HG are predominantly aqueous in nature, so it is expected that they will exhibit a similar release for a drug with high water solubility such as ETVC, as observed in our results (Figure 2). The diffusion release of this drug would be easier from HG and water than from BG, as discussed in the previous section [64,68]. Furthermore, in the skin release tests (Figure 3), *P1* values suggested a higher tendency of ETVC to pass through the skin when applied with the HG compared to water solution. While membranes provide controlled environments for studying release kinetics, biological tissues like porcine skin introduce complexities related to skin interactions and permeation dynamics.

#### 3.2.2. Permeation into and through the Skin

**Figure 4:**
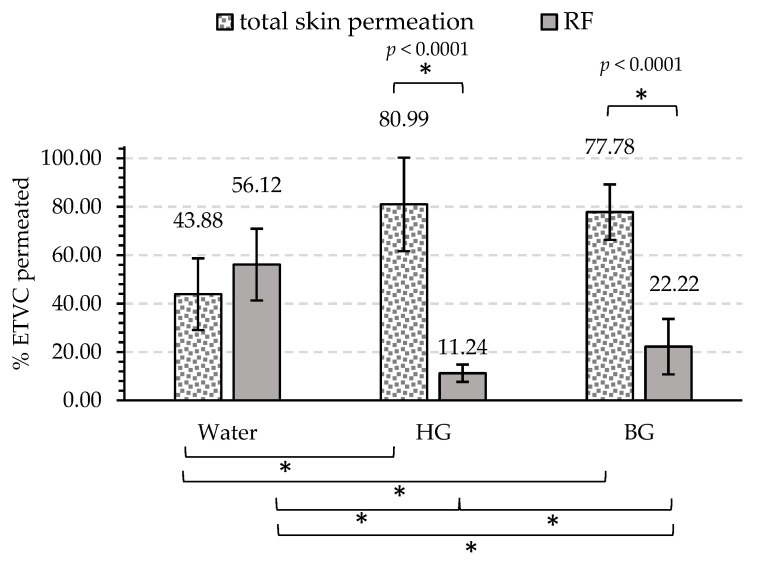
Percentage of ETVC permeated into the skin and receptor fluid after 24 h for the water solution, HG and BG. The sum of the amount of ETVC that permeated into the skin and RF is considered 100%. Significant differences are indicated with asterisks (*) (n = 6).

These results provide information about the tendency of ETVC to be retained in the skin or to pass into the receptor fluid depending on the applied material. In Figure 4, the percentage of total ETVC retained in the skin and permeated into the RF within 24 h for each sample is represented. The percentage data were calculated based on the total amount of ETVC that permeated into the skin and RF. All data values represented in Figure 4 and the statistical analyses are presented in Appendix C, Table A7 and Table A8, respectively.

Regarding skin permeation results, Figure 4 shows that after 24 h, ETVC is mostly retained in the skin when it is applied with the HG and the BG, obtaining 81% and 78%, respectively. Only 44% ETVC was retained in the skin when applied in aqueous solution. Significant differences were encountered in ETVC skin content between water–HG and water–BG (*p* < 0.01), but there were no significant differences between HG and BG. In RF results, water had the highest permeation, with 56% ETVC, followed by the BG and the HG, with 22% and 11%, respectively. Significant differences were encountered between all materials. These results correlate with results of kinetic release in the skin (Section 3.2.1), where including ETVC in delivery systems with lipid components (HG and BG) improves the interaction with the SC, promoting its retention in the skin. These results indicate that carrying a hydrophilic molecule in a material composed of lipid nanostructures, mainly phospholipids, favors its retention in the skin. It seems that HG has a relevant role in this retention, since the behavior of BG was similar despite having a higher lipid content.

#### 3.2.3. ETVC Distribution into Skin Layers

**Figure 5:**
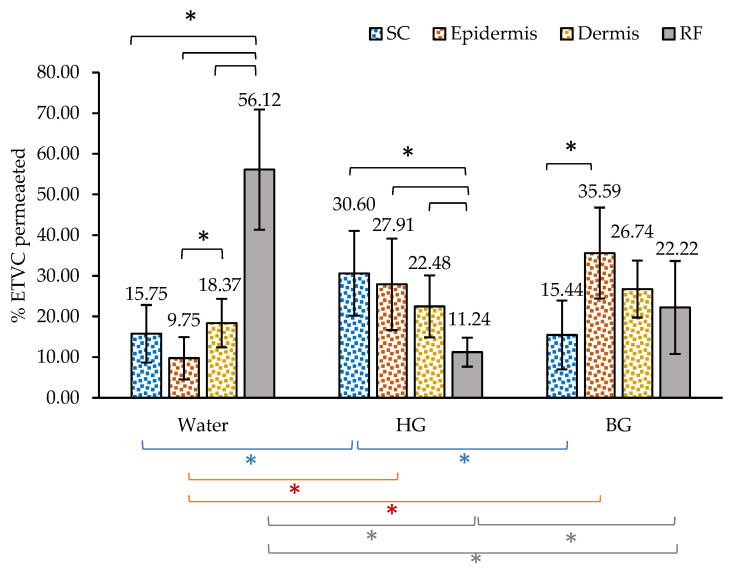
Percentage of ETVC retained in the skin layers and permeated into the RF after 24 h for aqueous solution, HG and BG 10% ETVC. The sum of the amount of ETVC that permeated into the skin and RF is considered 100%. Significant differences between layers for each individual material are shown with black asterisks (*). Permeation differences between materials are shown with blue asterisks for SC (*), orange asterisks for epidermis (*) and gray asterisks for RF (*) (n = 6).

These results provide information about the potential of each material to deliver ETVC into specific skin layers. Figure 5 clearly illustrates the distribution of ETVC across the stratum corneum (SC), epidermis, dermis and RF after 24 h. The percentage data were calculated based on the total amount of ETVC that permeated into the skin and RF. All data values represented in Figure 5 and the statistical analyses are presented in Appendix C, Table A9, Table A10 and Table A11, respectively. Significant differences between layers of each material are represented with black asterisks. Significant differences between materials are depicted in Figure 5, with blue asterisks for SC, orange asterisks for epidermis and gray asterisks for RF.

Regarding the permeated ETVC after application in water solution, the following differences were observed: When ETVC was applied in aqueous solution, 56% of the drug was detected in the RF. Additionally, the dermis contained a higher ETVC content (18.37%) than the epidermis (9.75%) (see black asterisks in Figure 5). On the other hand, when HG and BG were applied, a higher quantity of ETVC was retained in the skin layers (as shown in previous Figure 4). There is a tendency for the ETVC applied with HG to be retained in the SC, followed by the epidermis. Regarding BG results, the ETVC content in the epidermis (35.59%) was double the SC ETVC content (15.44%), so there seems to be a tendency for the BG to introduce ETVC into deeper layers.

Permeation results in the skin (Figure 4 and Figure 5) provide important insights into the retention of ETVC in the skin depending on the material. The water sample showed greater transdermal penetration, with a higher quantity of ETVC found in the RF. In contrast, the HG and BG formulations exhibited an enhanced retention of ETVC within the skin layers. According to the literature [68,69,70], the introduction of phospholipids in a drug delivery system enhances the interaction with SC lipids, facilitating the penetration of the skin, which agrees with our results. The HG retains ETVC predominantly within the superficial layers of the skin, offering antioxidant benefits to areas exposed to pollutants. Banov et al. [69] reported a similar tendency, where applying a material with phospholipids led to the majority of a certain drug being retained in the SC, and Folle et al. [71] also reported that nanoparticles loaded with phospholipids penetrated the primary layers of the epidermis. In contrast, the BG formulation facilitates the deeper penetration of ETVC into the epidermis and dermis, potentially extending its protective effects to deeper skin layers. These results are consistent with previous studies by Loza-Rodríguez et al. [34], where a hydrophilic probe penetrated more into the epidermis when a BG was applied compared to an HG. The interaction of the BG with the skin could be attributed to its richer lipid composition. As reported in previous studies [34], incorporating HG into an OG matrix increases the lipophilic nature of the material compared to HG, which likely enhances the BG interaction with the skin [46,72]. The OO present in the OG could act as a permeation enhancer, as described by Mahmood et al. [73]. Additionally, the fatty acids and fatty acid esters in BW could interact with the ceramides of the SC, enhancing the permeation of this skin layer, as reported by Fratini et al. and Meckfessel et al. [74,75].

Even though in the release skin kinetics showed no differences between the HG and the BG, in this section, in Figure 5, we clearly see a tendency of ETVC to permeate the deeper layers with the BG, whereas in the HG, ETVC is primarily found in the outermost layers of the skin. Receptor fluid results are the same as those in Section 3.2.2, which have been already discussed. Furthermore, even though the in vitro release studies (Section 3.1) indicated that the release behavior of the BG was lower than that of the HG, in this section, we observe a higher quantity of ETVC in the RF for the BG when applied to the skin compared to the HG, indicating a higher bioavailability of ETVC in the skin when the BG is applied.

These permeation results suggest that the HG and BG could be strategically utilized based on specific therapeutic or cosmetic targets. They would be suitable to deliver ETVC into the skin, particularly for a topical treatment. HG would provide ETVC release into the outermost layers of the skin, while the BG could be more effective to introduce higher amounts of ETVC into deeper layers, which may be of potential value to provide adequate antioxidant properties to skin at risk of oxidative or inflammatory damage. For example, the gels could be used to deliver vitamin C to aged and photoaged skin because vitamin C concentration decreases due to prolonged exposure to oxidative stress.

### 3.3. Skin Surface Lipid Structure

**Figure 6:**
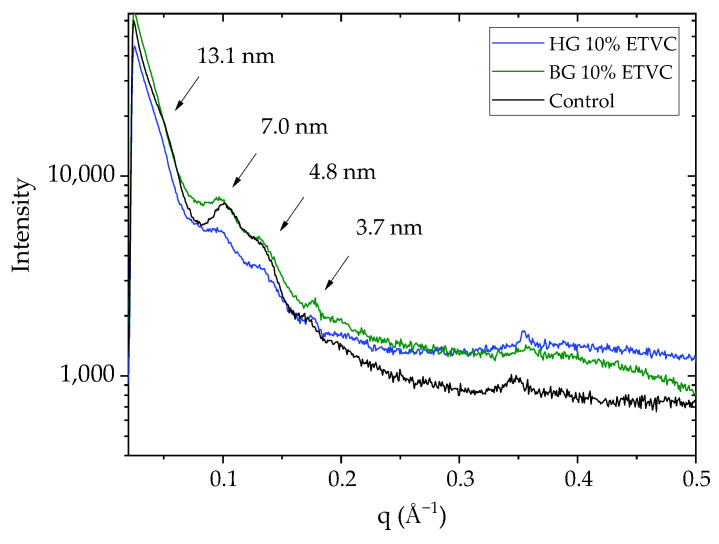
Grazing incidence small-angle X-ray scattering (GISAXS) intensity profiles of the skin treated with HG 10% ETVC and BG 10% ETVC and a skin control treated with water.

Small-angle X-ray scattering provides information about the larger structural units in the sample, such as the repeat distances (d-spacing) of a lamellar phase, which were related to the lamellar lipid structure in studies of the SC [76,77,78]. Due to the low quantity of lipids in the SC, the grazing incidence small-angle X-ray scattering (GISAXS) technique was utilized. This is particularly useful for thin samples because the skin surface is aligned at a specific angle with the X-ray beam to achieve a favorable path length. Consequently, despite the thinness of the sample, the intensity signal is strong when the path length is increased. In this study, pig skin was employed to assess whether the application of HG and BG modified the structure of the SC or not. Figure 6 shows the GISAXS intensity profiles corresponding to the control and treated samples with HG and BG.

The pattern exhibits a subtle change in slope at q = 0.048 Å^−1^, corresponding to a distance of 13.1 nm. Subsequently, two reflections at q = 0.09 Å^−1^ and q = 0.13 Å^−1^ are observed, corresponding to repeat distances of 7.0 nm and 4.8 nm, which may represent the second and third reflections of the 13.1 nm reflection, respectively. Additionally, a fourth reflection is visible at q = 0.17 Å^−1^, corresponding to a distance of 3.7 nm. 

**Figure A2:**
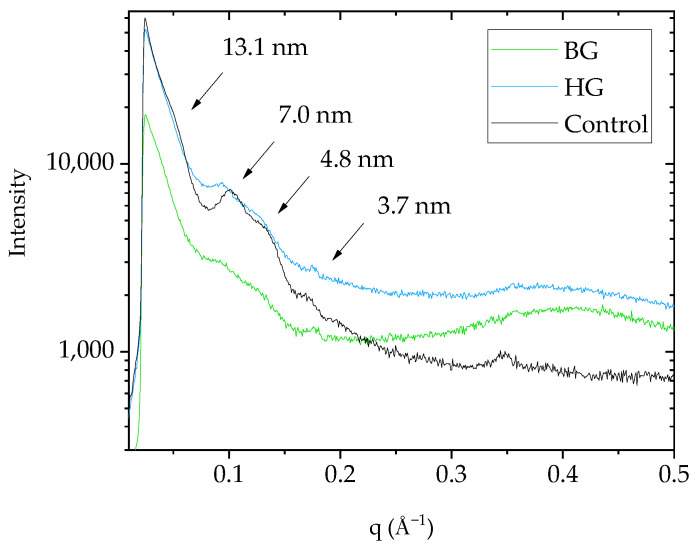
Grazing incidence small-angle X-ray scattering (GISAXS) intensity profiles of the skin treated with HG and BG without ETVC.

Overall, the profiles of the skin control treated with water and the skin treated with samples exhibit similar peaks and profiles, with no increase in the intensity of the reflections or a shift towards larger or smaller q values. Our results agree with those published by Rodríguez et al. [76,77], Jager et al. [78] and Moner [79]. All these reflections correspond to the different lamellar structures of the SC. According to Jager et al. [78], the lipids of native SC are organized in two lamellar phases, one with a repeat distance around 13.5 nm, which corresponds to the large lamellar phase (LLP), and the other one with a repeat distance around 6 nm, which corresponds to the short lamellar phase (SLP). In our results, the SLP reflection is observed around 7 nm (see Figure 6). The reflection at 4.8 nm could correspond to the third order of the LLP reflection (13.1 nm). The reflection around 3.7 of low intensity could correspond to crystalline cholesterol [80] or to the fourth order of the LLP at d = 13.1 nm [76,77]. In Appendix D, Figure A2, the GISAXS graph of the skin treated with the lipidic gels without ETVC also shows the same peaks as in Figure 6. The GISAXS profiles are not altered by either the ETVC delivery or the lipidic gels compared to the control treated with water. Therefore, HG and BG promote ETVC permeation in different layers of the skin without altering the lamellar structure of the SC, thus maintaining the integrity of the skin barrier function. We hypothesize that the lipids of our HG and BG formulations may interact with the intercellular lipids of the bilayer and lamellar structures of the SC, causing a non-permanent alteration in the laminar structure and enhancing the ETVC to permeate through the different possible transepidermal permeation routes [67] and then be retained in the different layers of the skin. This alteration may occur while the gel is in contact with the skin. Once the gels are removed from the top of the skin, the SC would be fully recovered to its normal barrier properties, maintaining the integrity of the skin barrier function [67].

### 3.4. In Vitro Skin Antioxidant Efficacy

**Figure 7:**
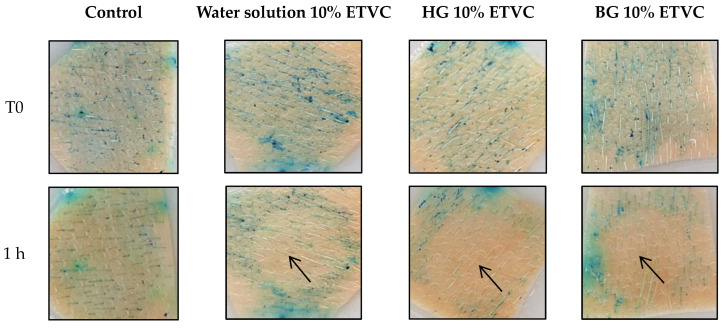
Images showing the in vitro antioxidant activity of aqueous solution 10% ETVC, HG 10% ETVC, BG 10% ETVC and water control after one hour of application. The reduction of methylene blue to colorless leucomethylene blue (colorless areas indicated by arrows) shows the qualitative antioxidant capacity of the materials on the skin surface.

The antioxidant activity of the HG, BG and water solution with 10% ETVC were assessed using in vitro pig skin incubated for 1 h. In Figure 7, results are represented as images before and after the treatment.

All materials tested showed antioxidant activity; this can be observed in the colorless areas 1 h after the treatment (see arrows in Figure 7). Fernández-García et al. [63] reported this reaction of methylene blue in reduction to colorless leucomethylene blue, showing the qualitative antioxidant capacity of the materials compared to the control. A slightly higher activity can be observed for HG and BG when compared qualitatively to that of the water solution. Folle et al. [81] also reported the antioxidant activity of their nanoparticles using the same experiment with porcine skin as a preliminary antioxidant efficacy result. Thus, it can be predicted that ETVC incorporated in the HG and the BG would show improved antioxidant activity in the skin. According to Pullar et al. [3], ascorbic acid derivatives for the purpose of topical applications must be converted to AA in vivo in order to be effective. In this case, this is not an in vivo study, but the actual antioxidant molecule is AA, resulting from ETVC conversion in the skin.

## 4. Conclusions

This work studies the ETVC release, the permeation behavior and the skin interaction of an HG and a BG, which are made only with lipids and water without the intervention of any polymeric compound. The differences observed in release kinetics between dialysis membranes and in vitro skin permeation studies highlight the impact of formulation composition. The HG, characterized by dispersed lipid vesicles, exhibited a rapid release. The BG enabled controlled and sustained ETVC release over time due to its double encapsulation structure but demonstrated a more rapid kinetic trend in skin permeation studies compared to the HG. To conclude, the incorporation of lipids into these materials plays a crucial role in promoting ETVC retention within skin layers without compromising the laminar structure of the SC. Both HG and BG exert an antioxidant effect on the skin with the permeation of hydrophilic molecules like ETVC while preserving skin barrier function, suggesting their strategic potential for specific therapeutic or cosmetic targets.

## Figures and Tables

**Table 1:** Composition of the HGs and BG.

Material	Hydrogel 25% ETVC	Hydrogel 10% ETVC	Bigel 10% ETVC
	%*w*/*w*	%*w*/*w*	%*w*/*w*
Water	70	85	28
HSPC	4.8	4.8	1.9
DOTAP	0.2	0.2	0.1
ETVC	25	10	10
OO	-	-	53.4
BW	-	-	6
α-Tocopherol	-	-	0.6

**Table 2:** Kinetic parameters of ETVC release for the different materials (obtained following Equation (1)). Lower *Kd* values mean higher velocities (see Equation (1)).

Material	R^2^	*Bmax* ± SD (% ETVC)	*Kd* ± SD (h)
Water solution 10% ETVC	0.998	79.91 ± 4.08	0.382 ± 0.15
HG 10% ETVC	0.997	90.35 ± 7.33	1.16 ± 0.37
BG 10% ETVC	0.992	60.90 ± 8.44	1.636 ± 0.76

**Table 3:** Kinetic skin parameters of ETVC in porcine skin for water solution, HG and BG. *J* is the permeation rate at steady state, *Tl* is the lag time, *Kp* is the permeability constant, *P1* is related with the partition coefficient of the ETVC vehicle skin and *P2* is related to the diffusion coefficient. Results are represented as the median and its minimum and maximum values.

Material	R^2^	*J* (µg/cm^2^·h)	*Tl* (h)	*Kp* (cm/h)	*P1* (cm)	*P2* (h^−1^)
Water solution 10% ETVC	0.999	10.33 (3.56–17.08)	14.83 (11.38–18.15)	1.09 × 10^−3^ (0.38–1.80)·10^−3^	0.40 × 10^−3^ (0.20–0.60)·10^−3^	2.47 (1.90–3.03)
HG 10% ETVC	0.999	4.61 (1.46–7.77)	12.65 (12.51–12.72)	0.46 × 10^−3 a^ (0.14–0.77)·10^−3^	0.22 × 10^−3 a^ (0.07–0.36)·10^−3^	2.11 (2.09–2.12)
BG 10% ETVC	0.999	8.45 (3.97–10.43)	12.73 (11.27–13.56)	0.78 × 10^−3^ (0.37–0.96)·10^−3^	0.41 × 10^−3^ (0.17–0.43)·10^−3^	2.12 (1.88–2.26)

^a^ means significant differences from water solution 10% ETVC.

## Data Availability

The data presented in this study are available on request from the corresponding author. The data are not publicly available due to privacy restrictions.

## Appendix A. In Vitro Release Studies

**Table A1:** In vitro kinetic studies results, extraction time and data represented in Figure 2.

Water Solution 10% ETVC			HG 10% ETVC			BG 10% ETVC		
Extraction Time (h)	Mean (% ETVC)	SD	Extraction Time (h)	Mean (% ETVC)	SD	Extraction Time (h)	Mean (% ETVC)	SD
0.0	0.00	0.00	0.0	0.00	0.00	0	0.00	0.00
0.5	60.24	12.78	1.0	45.94	4.53	1	26.72	12.37
1.5	64.73	10.60	2.0	58.75	6.70	2	36.20	2.27
2.5	71.62	6.65	3.0	61.55	6.26	3	38.10	4.48
3.5	71.22	7.80	4.0	66.96	6.07	4	40.70	8.98
4.5	71.79	9.36	5.0	71.03	2.27	5	40.89	12.36
24.0	82.10	8.81	24.0	90.14	4.91	24	60.80	13.42

**Table A2:** Two-way ANOVA results of the in vitro release studies (see Figure 2). Data obtained from GraphPad Prism 9.

Time Point	Material Interaction	Q Value	Individual *p* Value	Significant Differences
1 h	HG–water	0.0228	0.0653	Yes
	BG–water	0.0042	0.0040	Yes
	BG–HG	0.081	0.0155	Yes
2 h	HG–water	0.1038	0.2966	No
	BG–water	0.0023	0.0044	Yes
	BG–HG	0.0017	0.0016	Yes
3 h	HG–water	0.0374	0.0356	Yes
	BG–water	0.0007	0.0002	Yes
	BG–HG	0.0051	0.0032	Yes
4 h	HG–water	0.1168	0.3338	No
	BG–water	0.0012	0.0011	Yes
	BG–HG	0.0012	0.0023	Yes
5 h	HG–water	0.3003	0.8579	No
	BG–water	0.0007	0.0014	Yes
	BG–HG	0.0007	0.0010	Yes
24 h	HG–water	0.0392	0.1121	Yes
	BG–water	0.0048	0.0091	Yes
	BG–HG	0.0005	0.0005	Yes

**Table A3:** One-way ANOVA results of the *Bmax* and *Kd* parameters from the in vitro release studies.

Parameter	Material Interaction	Q Value	Individual *p* Value	Significant Difference
*Bmax*	HG–water	3.04	*p* > 0.05	No
	BG–water	5.53	*p* < 0.01	Yes
	BG–HG	8.57	*p* < 0.001	Yes
*Kd*	HG–water	3	*p* > 0.05	No
	BG–water	5	*p* < 0.05	Yes
	BG–HG	2	*p* > 0.05	No

## Appendix B. In Vitro Skin Kinetic Studies

**Table A4:** In vitro skin kinetic studies, extraction time and data represented in Figure 3.

Water Solution 10% ETVC			HG 10% ETVC			BG 10% ETVC		
Extraction Time (h)	Mean % ETVC	SD	Extraction Time (h)	Mean % ETVC	SD	Extraction Time (h)	Mean % ETVC	SD
0.0	0.00	0.00	0.0	0	0	0.0	0	0
1.2	0.00	0.00	1.2	0.00	0.00	1.2	0.02	0.01
2.7	0.00	0.00	2.7	0.03	0.01	2.7	0.10	0.05
4.2	0.01	0.00	4.2	0.05	0.02	4.2	0.18	0.10
19.9	0.58	0.02	19.9	0.50	0.43	19.9	0.90	0.36
22.0	1.00	0.70	22.0	0.65	0.50	22	1.10	0.40
25.3	1.68	1.00	25.6	0.87	0.75	26.0	1.56	0.62

**Table A5:** Two-way ANOVA results of the in vitro kinetic studies (see Figure 3). Data obtained from Graphpad Prism 9.

Time Point	Material Interaction	Q Value	Individual *p* Value	Significant Difference
1 h	HG–water	0.1611	0.1534	No
	BG–water	0.0264	0.0126	Yes
	BG–HG	0.5708	0.8154	No
3h	HG–water	0.0836	0.0796	No
	BG–water	0.0092	0.0044	Yes
	BG–HG	0.6687	0.9552	No
4 h	HG–water	0.1012	0.0964	No
	BG–water	0.0109	0.0052	Yes
	BG–HG	0.6011	0.8586	No
20 h	HG–water	0.3470	0.2203	No
	BG–water	0.1998	0.0634	No
	BG–HG	0.4612	0.4392	No
22 h	HG–water	0.5619	0.3568	No
	BG–water	0.7497	0.7140	No
	BG–HG	0.1550	0.0492	No
24 h	HG–water	0.7702	0.4890	No
	BG–water	0.8388	0.7968	No
	BG–HG	0.7702	0.4132	No

**Table A6:** One-way ANOVA results of the in vitro kinetic studies (see Table 3). Data obtained from Graphpad Prism 9.

Kinetic Parameter	Material Interaction	Q Value	Individual *p* Value	Significant Difference
*J* (µg/cm^2^·h)	HG–water	0.0808	0.0513	No
	BG–water	0.9017	0.8588	No
	BG–HG	0.0653	0.0207	No
*Tl* (h)	HG–water	0.6215	0.3277	No
	BG–water	0.6215	0.5919	No
	BG–HG	0.6215	0.5919	No
*Kp* (cm/h)	HG–water	0.0298	0.0142	**Yes**
	BG–water	0.3316	0.4738	No
	BG–HG	0.0513	0.0488	No
*P1* (cm)	HG–water	0.0300	0.0143	**Yes**
	BG–water	0.3320	0.4743	No
	BG–HG	0.0516	0.0491	No
*P2* (h^−1^)	HG–water	0.7415	0.4589	No
	BG–water	0.7415	0.4708	No
	BG–HG	0.9746	0.9282	No

## Appendix C. In Vitro Permeation Studies

**Table A7:** ETVC distribution into skin layers, data represented in Figure 4.

Material	Total Skin Permeation	Receptor Fluid
Water solution 10% ETVC	43.88 ± 14.80	56.12 ± 14.80
HG 10% ETVC	80.99 ± 19.30	11.24 ± 3.57
BG 10% ETVC	77.78 ± 11.42	22.22 ± 11.42

**Table A8:** One-way ANOVA results of the in vitro total skin permeation studies (see Figure 4). Differences between ETVC content for RF and skin, respectively. Data obtained from Graphpad Prism 9.

One-Way ANOVA	Material Interaction	Q Value	Individual *p* Value	Significant Difference
RF	HG–water	0.0019	0.0019	Yes
	BG–water	0.0019	0.0035	Yes
	BG–HG	0.0233	0.0665	Yes
Total skin permeation	HG–water	0.0030	0.0057	Yes
	BG–water	0.0030	0.0035	Yes
	BG–HG	0.2572	0.7348	No

**Table A9:** ETVC distribution in skin layers, data represented in Figure 5.

Material	Stratum Corneum	Epidermis	Dermis	Receptor Fluid
Water solution 10% ETVC	15.75 ± 7.07	9.75 ± 5.20	18.37 ± 5.96	56.12 ± 14.80
HG 10% ETVC	30.60 ± 10.42	27.91 ± 11.26	22.48 ± 7.62	11.24 ± 3.57
BG 10% ETVC	15.44 ± 8.47	35.59 ± 11.18	26.74 ± 7.00	22.22 ± 11.42

**Table A10:** One-way ANOVA results of the in vitro permeation studies through skin layers. Differences between ETVC content in skin layers for each material (see Figure 5). Data obtained from Graphpad Prism 9.

Material	Skin Layer Interaction	Q Value	Individual *p* Value	Significant Difference
Water solution 10% ETVC	SC–epidermis	0.1061	0.1684	No
	SC–dermis	0.2859	0.5445	No
	SC–RF	0.0028	0.0018	Yes
	Epidermis–dermis	0.0325	0.0413	Yes
	Epidermis–RF	0.0028	0.0012	Yes
	Dermis–RF	0.0029	0.0028	Yes
HG 10% ETVC	SC–epidermis	0.3549	0.6760	No
	SC–dermis	0.1237	0.1571	No
	SC–RF	0.0145	0.0047	Yes
	Epidermis–dermis	0.2232	0.3543	No
	Epidermis–RF	0.0145	0.0135	Yes
	Dermis–RF	0.0145	0.0138	Yes
BG 10% ETVC	SC–epidermis	0.0325	0.0062	Yes
	SC–dermis	0.0820	0.0312	No
	SC–RF	0.2860	0.2724	No
	Epidermis–dermis	0.1801	0.1372	No
	Epidermis–RF	0.1185	0.0677	No
	Dermis–RF	0.3777	0.4317	No

**Table A11:** Two-way ANOVA results of the in vitro permeation studies through skin layers. Differences in the interaction between materials for each skin layer (see Figure 5).

Time Point	Material Interaction	Q Value	Individual *p* Value	Significant Difference
SC	HG–water	0.0112	0.0213	Yes
	BG–water	0.3320	0.9486	No
	BG–HG	0.0112	0.0207	Yes
Epidermis	HG–water	0.0047	0.0090	Yes
	BG–water	0.0014	0.0013	Yes
	BG–HG	0.0921	0.2631	No
Dermis	HG–water	0.3602	0.3431	No
	BG–water	0.1922	0.0610	No
	BG–HG	0.3602	0.3368	No
RF	HG–water	0.0019	0.0018	Yes
	BG–water	0.0019	0.0035	Yes
	BG–HG	0.0233	0.0665	Yes
